# Stochastic Entropy Production for Classical and Quantum Dynamical Systems with Restricted Diffusion

**DOI:** 10.3390/e27040383

**Published:** 2025-04-03

**Authors:** Jonathan Dexter, Ian J. Ford

**Affiliations:** Department of Physics and Astronomy, University College London, Gower Street, London WC1E 6BT, UK

**Keywords:** stochastic thermodynamics, stochastic entropy production, open quantum system dynamics

## Abstract

Modeling the evolution of a system using stochastic dynamics typically implies increasing subjective uncertainty in the adopted state of the system and its environment as time progresses, and stochastic entropy production has been developed as a measure of this change. In some situations, the evolution of stochastic entropy production can be described using an Itô process, but mathematical difficulties can emerge if diffusion in the system phase space happens to be restricted to a subspace of a lower dimension. This situation can arise if there are constants of the motion, for example, or more generally when there are functions of the coordinates that evolve without noise. More simply, difficulties can emerge if there are more coordinates than there are independent noises. We show how the problem of computing the stochastic entropy production in such a situation can be overcome. We illustrate the approach using a simple case of diffusion on an ellipse. We go on to consider an open three-level quantum system modeled within a framework of Markovian quantum state diffusion. We show how a nonequilibrium stationary state of the system, with a constant mean rate of stochastic entropy production, can be established under suitable environmental couplings.

## 1. Introduction

Entropy quantifies subjective uncertainty in the configuration of a system when limited information is available. If the evolution of such a system is modeled using stochastic dynamics, for example, representing the effects of coupling to an underspecified environment, then uncertainty in the configuration of the world (the system plus its environment) increases with time, corresponding to growth in the total entropy. Entropy growth does not *control* the evolution of the world in a causal sense: how could a subjective uncertainty do such a thing? But, it does *characterize* its evolution. The second law of thermodynamics arises naturally when the world is perceived at a coarse-grained level but governed by underlying equations of motion with a certain degree of deterministic chaos [1].

For system coordinates that evolve continuously according to a set of Markovian stochastic differential equations (SDEs) or Itô processes, it may be shown that the associated stochastic entropy production [2,3,4] can also be described using an SDE, but only if the noise terms satisfy certain requirements [5]. Problems arise from constraints imposed on the dynamics, for example, the existence of constants of the motion. In such cases, the matrix describing the diffusion of system coordinates in their phase space becomes singular. There are directions in the space in which diffusion does not take place and mathematical difficulties in the evaluation of the stochastic entropy production arise as a consequence.

The central aim of this paper is to show how to take into account such constraints on diffusion when computing the stochastic entropy production. In Section 2, we briefly discuss how a Markovian SDE for stochastic entropy production may be derived from Markovian SDEs describing the evolution of a set of system coordinates. The mathematical treatment of cases where diffusion is restricted is discussed in Section 3. Application to a simple example case involving the stochastic evolution of two (classical) coordinates under the influence of just one noise is covered in Section 4. Situations where the number of stochastic variables exceeds the number of independent noises are commonplace in dynamical modeling and can lead to singular diffusion matrices.

In Section 5, we consider the stochastic dynamics of the reduced density matrix of a more complicated open three-level quantum system subjected to environmental disturbance that promotes transitions between the levels. The theoretical background to such situations has been developed recently and is discussed at greater length in [6,7]. The essential details are provided in Appendix B. The goal of such modeling is to identify the increase in uncertainty of the quantum state of a system and its environment arising from an uncontrolled and therefore indeterminate process of evolution, and stochastic entropy production provides such a characterization. The three-level system is one of the simplest that can sustain a nonequilibrium stationary state, a feature that we are interested in exploring. The diffusion matrix in the relevant parameter space is singular, but we demonstrate how the stochastic entropy production can still be evaluated. We go on to compute the environmental component of mean stochastic entropy production numerically in order to characterize the equilibrium and nonequilibrium stationary states of the system. Our conclusions are outlined in Section 6.

## 2. Stochastic Entropy Production for Itô Processes

We consider a set of *N* coordinates $x\equiv (x_{1},x_{2},\cdots ,x_{N})$ that specify the configuration of a system and model their evolution using Markovian stochastic differential equations, or Itô processes, of the form

(1) $$dx_{i}=A_{i}(x)dt+\sum_{j=1}^{M}B_{ij}(x)dW_{j},$$

where $dW_{j}$ are independent Wiener increments. The coefficients $A_{i}$ and $B_{ij}$ are functions of the coordinates, but, in the interest of compactness, this notation is suppressed hereafter. For simplicity, we confine the discussion to coordinates with *even parity* under time reversal.

Defining an N×N diffusion matrix $D=\frac{1}{2}BB^{T}$, the Fokker–Planck equation for the probability density function (PDF) p(x,t) is given by

(2) $$\begin{array}{cc} \frac{\partial p}{\partial t} & =-\sum_{i}\frac{\partial }{\partial x_{i}}\left(A_{i}p\right)+\sum_{ij}\frac{\partial }{\partial x_{i}\partial x_{j}}\left(D_{ij}p\right),\\ \; & =-\sum_{i}\frac{\partial }{\partial x_{i}}\left(C_{i}p-D_{ij}\frac{\partial p}{\partial x_{j}}\right), \end{array}$$

with

(3) $$C_{i}=A_{i}-\sum_{j}\frac{\partial D_{ij}}{\partial x_{j}}.$$

The stochastic entropy production of the system and its environment, associated with the stochastic motion described by Equation (1), is a measure of irreversibility. It is the difference in probability between pairs of time-reversed sequences of events and defined by [2]

(4) $$\Delta s_{\mathrm{tot}}=\ln\left(\frac{p(x,t)T(x\to x^{\prime },\Delta t)}{p(x^{\prime },t+\Delta t)T(x^{\prime }\to x,\Delta t)}\right),$$

where $T(x\to x^{\prime },\Delta t)$ is a probability that the system should take a (forward) path from x to $x^{\prime }$ in a period Δt. Note that the PDF in the denominator is specified at time t+Δt: the time-reversed counterpart of the forward path is considered to start after the forward path has been completed.

The total stochastic entropy production is usually separated into system and environmental contributions, with increments given by

(5) $$d\Delta s_{\mathrm{tot}}=d\Delta s_{\mathrm{sys}}+d\Delta s_{\mathrm{env}},$$

with $d\Delta s_{\mathrm{sys}}=-d\ln p(x,t)$. The evolution of the environmental stochastic entropy production is governed by the SDE [8],

(6) $$\begin{array}{cc} d\Delta s_{\mathrm{env}} & =\sum_{ij}D_{ij}^{-1}C_{j}dx_{i}+\sum_{ijk}D_{ij}\frac{\partial (D_{ik}^{-1}C_{k})}{\partial x_{j}}dt,\\ \; & =\sum_{ij}D_{ij}^{-1}C_{j}\circ dx_{i}. \end{array}$$

The final form, employing Stratonovich rules of stochastic calculus, is compact, although it is more practical to employ the Itô form given in the first line. A more general expression suitable for systems with odd as well as even parity coordinates is given in Appendix A.

It may be shown that the average of $d\Delta s_{\mathrm{sys}}$ over all possible trajectories is related to the incremental change in Gibbs entropy of the system: $d\langle \Delta s_{\mathrm{sys}}\rangle =dS_{G}$, where $S_{G}(t)=-\int p(x,t)\ln p(x,t)dx$, to which boundary terms should be added in certain circumstances [7]. Computing the environmental stochastic entropy production, on the other hand, presents particular difficulties if the diffusion matrix is singular since the inverse matrix $D^{-1}$ is required in the above expression. This is the problem we wish to address here.

## 3. Defining Dynamical and Spectator Variables

### 3.1. Preliminaries

A singular diffusion matrix may be regarded as a consequence of having fewer independent noise terms than the number of coupled Itô processes. For example, diffusion might occur on a two-dimensional surface within a three-dimensional phase space of system coordinates if the motion is described by three Itô processes with only two independent Wiener increments. There is a direction at each point in the phase space in which there is no diffusive current, which makes the 3×3 diffusion matrix singular. These directions lie parallel to spatially dependent eigenvectors of the diffusion matrix with zero eigenvalues, to be referred to as null eigenvectors.

The obvious solution is to derive a reduced set of stochastic differential equations that describe the random evolution of, in this example, two coordinates on the surface with the third coordinate related deterministically to the other two. We shall denote the stochastically evolving coordinates as ‘dynamical variables’ and the remaining coordinates as ‘spectator variables’. It might be possible in simple cases to form such a reduced set of dynamical variables, perhaps by identifying a constant of the motion. However, as we increase the dimensionality of the phase space and hence the size of the diffusion matrix, the difficulties in doing so may become insurmountable. We therefore require a more general treatment of situations with a singular diffusion matrix.

So, let us consider a system described by *N* coordinates $x_{i}$, each of which evolves stochastically according to Equation (1). According to Itô’s lemma [9], the differential of a function *f* of these coordinates can be written

(7) $$df=\sum_{i=1}^{N}\frac{\partial f}{\partial x_{i}}dx_{i}+\sum_{i,j=1}^{N}\frac{\partial^{2}f}{\partial x_{i}\partial x_{j}}D_{ij}dt,$$

where the elements of the (symmetric) N×N diffusion matrix are $D_{ij}=\frac{1}{2}\sum_{k=1}^{M}B_{ik}B_{jk}$. Let us now consider a special case of a function where the stochastic terms in Equation (7) vanish, i.e.,

(8) $$\sum_{i=1}^{N}\sum_{j=1}^{M}\frac{\partial f}{\partial x_{i}}B_{ij}dW_{j}=0,$$

such that *f* evolves deterministically. Taking the square and using $dW_{i}dW_{j}=\delta_{ij}dt$, we find that

(9) $$\begin{array}{cc} \sum_{ijkl}\frac{\partial f}{\partial x_{i}}\frac{\partial f}{\partial x_{k}}B_{ij}B_{kl}\delta_{jl}dt & =\sum_{ijk}\frac{\partial f}{\partial x_{i}}\frac{\partial f}{\partial x_{k}}B_{ij}B_{kj}dt\\ \; & =2\sum_{ik}\frac{\partial f}{\partial x_{i}}\frac{\partial f}{\partial x_{k}}D_{ik}dt=0, \end{array}$$

showing that there is deterministic (noise-free) evolution of *f* if

(10) $${\left(\nabla f\right)}^{T}D\nabla f=0.$$

Such an outcome can arise if ∇f is an eigenvector of D with an eigenvalue equal to zero. A matrix is singular if one or more of its eigenvalues are zero, so we have established that D is singular if there exists a function *f* of the $x_{i}$ that evolves deterministically. For dynamics evolving in an *N*-dimensional phase space under the influence of *M* noises where N>M, we conjecture that there will be L=N−M such functions, each associated with one of the null eigenvectors of D. We assume that these functions and their associated null eigenvectors are mutually independent. We shall see that this allows us to recast the calculation of the stochastic entropy production to overcome the problematic singularity of D.

### 3.2. Identifying Constants of Motion

If the deterministic as well as stochastic terms in Equation (7) vanish, then df=0, and the function *f* is a constant of the motion; the evolution of the coordinates is constrained to a contour of constant *f*. We can therefore write

(11) ∇f·dx=0,

meaning that the infinitesimal vector dx specified by Equation (1) is tangential to a contour of *f*. Since ∇f is also a null eigenvector of the diffusion matrix, this constraint on dx is conveniently identified by evaluating the eigenvectors of D.

If there are L=N−M constant functions of the coordinates under the dynamics, we can remove *L* coordinates from the original *N*-dimensional phase space, leaving a reduced *M*-dimensional phase space. Without loss of generality, the first *M* coordinates in the set $\left\{x_{m}\right\}$ with m=1,⋯,M will be taken to be dynamical variables, and the remaining *L* coordinates $\left\{x_{l}\right\}$ with l=M+1,⋯,N are designated spectator variables. The *L* constants of the motion mean we should be able to write the spectator variables as functions of the dynamical variables, namely as $x_{l}\left(\left\{x_{m}\right\}\right)$. The division into dynamical and spectator variables is arbitrary, up to a point to be discussed shortly, but, as we shall see in Section 4, some choices are more convenient for computing stochastic entropy production than others.

In this labeling scheme, the top left M×M block of the diffusion matrix D remains relevant to the stochastic entropy calculation and will be nonsingular, allowing us to use Equation (6) to compute $\Delta s_{\mathrm{env}}$ associated with the evolution, but with *i* and *j* ranging only between 1 and *M* rather than 1 and *N*. If the elements of this matrix block depend on spectator variables, we need to take this into account when computing derivatives with respect to the dynamical variables. To emphasize this point, we write the components of A,D and $D^{-1}$ that appear in Equation (6) to show their dependence on the dynamical and spectator variables explicitly as follows:

(12) $$\begin{array}{c} A_{i}(\left\{x_{m}\right\},\left\{x_{l}(\left\{x_{m}\right\})\right\})\\ D_{ij}(\left\{x_{m}\right\},\left\{x_{l}(\left\{x_{m}\right\})\right\})\\ D_{ij}^{-1}(\left\{x_{m}\right\},\left\{x_{l}(\left\{x_{m}\right\})\right\}), \end{array}$$

and we note that Equation (6) requires us to evaluate derivatives of $A_{i}$, $D_{ij}$, and $D_{ij}^{-1}A_{j}$ with respect to the dynamical variables $\left\{x_{m}\right\}$.

We consider derivatives of $D_{ij}$ in the following, but the argument is easily extended to other expressions. We begin by noting that Equation (11) can be separated according to dynamical and spectator variables such that

(13) $$\sum_{m=1}^{M}\alpha_{km}dx_{m}+\sum_{l=M+1}^{N}\alpha_{kl}dx_{l}=0,$$

where $\alpha_{km}$ and $\alpha_{kl}$ are the dynamical and spectator components, respectively, of the *k*th null eigenvector of D, with k=1,⋯,L. Therefore,

(14) $$\sum_{l=M+1}^{N}\alpha_{kl}dx_{l}=-\sum_{m=1}^{M}\alpha_{km}dx_{m},$$

and, arranging the $\alpha_{km}$ as elements of a rectangular L×M matrix $Q_{km}$ and the $\alpha_{kl}$ as elements of a square L×L matrix $P_{kl}$, we have

(15) $$dx_{l}=-\sum_{k,m}P_{lk}^{-1}Q_{km}dx_{m}=\sum_{m=1}^{M}R_{lm}dx_{m},$$

where $R_{lm}$ is an element of the L×M matrix $R=-P^{-1}Q$. It should be noted that the invertibility of the P matrix would be threatened if the spectator components of the *L* null eigenvectors happen not to be independent, but this is not a situation we have encountered in example cases studied so far.

Next, we write

(16) $$dD_{ij}=\sum_{m=1}^{M}\frac{\partial D_{ij}}{\partial x_{m}}dx_{m}+\sum_{l=M+1}^{N}\frac{\partial D_{ij}}{\partial x_{l}}dx_{l},$$

and, by substituting $dx_{l}$ from Equation (15) into Equation (16), we arrive at the following expression for the required derivative of $D_{ij}$ with respect to the dynamical coordinate $x_{m}$:

(17) $$\frac{dD_{ij}}{dx_{m}}=\frac{\partial D_{ij}}{\partial x_{m}}+\sum_{l=M+1}^{N}\frac{\partial D_{ij}}{\partial x_{l}}R_{lm}.$$

The second term on the right-hand side provides an additional contribution to the dependence of $D_{ij}$ on $x_{m}$.

To summarize, the framework allows the computation of stochastic entropy production in cases where the diffusion matrix is singular due to constraints on the dynamics from the *L* constants of motion. The method employs these constants of motion to reduce the dimensionality of the phase space through which the system evolves such that the appropriately reduced diffusion matrix is nonsingular. Having carried out this transformation, Equation (6) can be used to compute the environmental component of stochastic entropy production with derivatives determined according to Equation (17).

### 3.3. Identifying Deterministically Evolving Functions

We have considered a function *f* of the stochastic variables $\left\{x_{i}\right\}$ that evolves according to

(18) $$df=\sum_{i}\frac{\partial f}{\partial x_{i}}dx_{i}+\sum_{i,j}\frac{\partial^{2}f}{\partial x_{i}\partial x_{j}}D_{ij}dt,$$

and looked at situations where both the deterministic and stochastic terms in Equation (18) vanish, making the function *f* a constant of motion of the dynamics. However, it is only necessary for the *stochastic* terms to vanish for there to be a restriction on the diffusive motion. We now consider the more general case where *f* evolves deterministically according to

(19) $$df=\left(\sum_{i}\frac{\partial f}{\partial x_{i}}A_{i}+\sum_{i,j}\frac{\partial^{2}f}{\partial x_{i}\partial x_{j}}D_{ij}\right)dt.$$

Following earlier arguments, as a consequence of Equation (8), ∇f is still a null eigenvector of D, and we now take each null eigenvector to correspond to a deterministically evolving function. To illustrate this, consider a system evolving stochastically in two dimensions ($x_{1}$,$x_{2}$) with a diffusion matrix that possesses a single null eigenvector. A function $f(x_{1},x_{2})$ evolving deterministically according to Equation (19) allows us to reduce the number of variables needed to describe the motion from two to one dynamical variable. The other becomes a spectator variable.

To understand this better, imagine that, at time $t_{0}$, the function *f* is given by

(20) $$f(x_{1}(t_{0}),x_{2}(t_{0}))=c_{0},$$

where $c_{0}$ is a constant defining a contour of *f* in the space. Equation (20) allows us to express spectator variable $x_{2}$ as a function of dynamical variable $x_{1}$ at time $t_{0}$.

Between times $t_{0}$ and $t_{1}=t_{0}+dt$, *f* changes deterministically by an amount df to define a new contour

(21) $$f(x_{1}(t_{1}),x_{2}(t_{1}))=c_{1}=c_{0}+df,$$

according to which we can again express $x_{2}$ as a function of $x_{1}$ at the later time. The evolution of the system is confined to a sequence of contours of the deterministically evolving function *f* such that we can always express $x_{2}$ in terms of $x_{1}.$ We can parameterize this evolution with a single coordinate and thereby employ a reduced diffusion matrix to compute a stochastic entropy production. The argument easily generalizes to an arbitrary number of dimensions.

**Figure 1:**
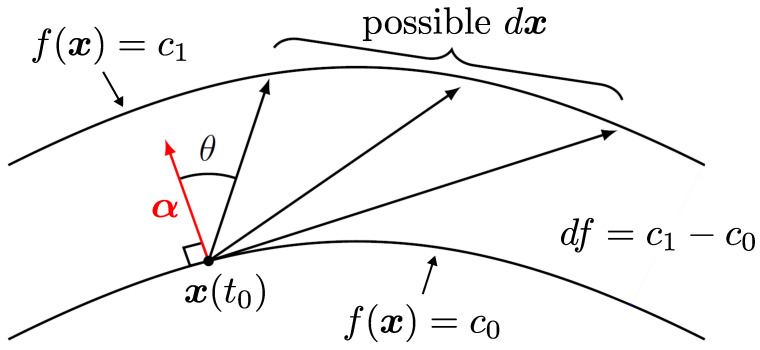
Illustration of the evolution of system coordinates between contours defined by a deterministically evolving function f(x), constructed to be normal to a spatially dependent null eigenvector α of the diffusion matrix. There is a restricted set of possible stochastic increments dx, defined by angle θ and the contours visited at the beginning and end of the timestep, which constrains the diffusive evolution and complicates the computation of the stochastic entropy production.

As in the previous section, we need to consider how derivatives are modified when we reduce the dimensionality of the phase space. We write ∇f=gα, where *g* is equal to |∇f| and α is a normalized null eigenvector of D, with |α|=1, that lies perpendicular to the contour of *f*. Infinitesimal changes in coordinates x over a timestep dt are given by dx, and x is constrained to move between points on specified contours of *f* at specified times, hence making only certain dx allowed. The situation is illustrated in Figure 1.

The component of dx in the direction normal to *f* is given by

(22) $$dx_{⊥}=|dx|\cos\theta =|\alpha ||dx|\cos\theta =\alpha \cdot dx,$$

where the angle θ is shown in Figure 1. We can also write

(23) $$df=|\nabla f|dx_{⊥}=\left|\nabla f\right|\alpha \cdot dx=g\alpha \cdot dx,$$

where g=|∇f|, such that

(24) $$g\alpha \cdot dx=\left(\sum_{i}\frac{\partial f}{\partial x_{i}}A_{i}+\sum_{i,j}\frac{\partial^{2}f}{\partial x_{i}\partial x_{j}}D_{ij}\right)dt.$$

For notational convenience, we rewrite the term in brackets on the right-hand side as gG so that

(25) α·dx=Gdt.

For a set of null eigenvectors $\alpha_{k}$ of D labeled by k=1,…,L, there would be *L* deterministically evolving functions of the coordinates, and we can specify relationships between increments in dynamical and spectator variables such that

(26) $$\sum_{m=1}^{M}\alpha_{km}dx_{m}+\sum_{l=M+1}^{N}\alpha_{kl}dx_{l}=G_{k}dt.$$

Following the reasoning in Equation (15), we write

(27) $$\begin{array}{cc} dx_{l} & =-P_{lk}^{-1}Q_{km}dx_{m}+P_{lk}^{-1}G_{k}dt=R_{lm}dx_{m}+S_{l}dt, \end{array}$$

with implied summation over repeated indices, and, substituting Equation (27) into Equation (16), we obtain

(28) $$dD_{ij}=\sum_{m=1}^{M}\frac{\partial D_{ij}}{\partial x_{m}}dx_{m}+\sum_{l=M+1}^{N}\frac{\partial D_{ij}}{\partial x_{l}}\left(R_{lm}dx_{m}+S_{l}dt\right).$$

As before, the required derivatives of the relevant elements of the diffusion matrix take the form

(29) $$\frac{dD_{ij}}{dx_{m}}=\frac{\partial D_{ij}}{\partial x_{m}}+\sum_{l}\frac{\partial D_{ij}}{\partial x_{l}}R_{lm}.$$

We therefore find that contributions to derivatives with respect to dynamical variables, where the expression in question also depends on spectator variables, are the same whether *f* is a constant of the motion or a function evolving without noise. This is useful since, in general, we are unlikely to be able to determine if the singularity of a diffusion matrix is due to the existence of a constant function or one that evolves deterministically.

## 4. Example of Restricted Diffusive Evolution

To illustrate the above reasoning, consider the two SDEs

(30) $$\begin{array}{c} dx_{1}=(1-x_{1}^{2})dW\\ dx_{2}=-\frac{1}{2}x_{2}dt-x_{1}x_{2}dW, \end{array}$$

which represent a case of restricted diffusive evolution since there are two Itô processes but only one noise. The 2×2 diffusion matrix for the $\left(x_{1},x_{2}\right)$ phase space is

(31) $$\begin{array}{cc} \; & \frac{1}{2}\left(\begin{array}{cc} 1-x_{1}^{2} & 0\\ -x_{1}x_{2} & 0 \end{array}\right)\left(\begin{array}{cc} 1-x_{1}^{2} & -x_{1}x_{2}\\ 0 & 0 \end{array}\right)\\ \; & =\frac{1}{2}\left(\begin{array}{cc} {\left(1-x_{1}^{2}\right)}^{2} & -x_{1}x_{2}(1-x_{1}^{2})\\ -x_{1}x_{2}(1-x_{1}^{2}) & x_{1}^{2}x_{2}^{2} \end{array}\right), \end{array}$$

which is singular since the determinant is zero. We now compute the stochastic entropy production using Equations (4) and (5). The deterministic terms are $A_{1}=0$ and $A_{2}=-\frac{1}{2}x_{2}$. We proceed first by regarding $x_{1}$ as the dynamical variable and $x_{2}$ as a spectator and write

(32) $$\begin{array}{cc} & d\Delta s_{\mathrm{tot}}=-d\ln p_{1}(x_{1},t)+\frac{1}{D_{11}}A_{1}dx_{1}-\frac{1}{D_{11}}\frac{dD_{11}}{dx_{1}}dx_{1}\\ \; & +\left[D_{11}\frac{d}{dx_{1}}\left(\frac{1}{D_{11}}A_{1}\right)-D_{11}\frac{d}{dx_{1}}\left(\frac{1}{D_{11}}\frac{dD_{11}}{dx_{1}}\right)\right]dt, \end{array}$$

where $p_{1}(x_{1},t)=\int p(x_{1},x_{2},t)dx_{2}$ and $p(x_{1},x_{2},t)$ satisfies the Fokker–Planck equation associated with Equation (30). Since $A_{1}=0$ and $D_{11}=\frac{1}{2}{\left(1-x_{1}^{2}\right)}^{2}$, we can take derivatives unencumbered by implicit dependence on $x_{1}$ arising from dependence on $x_{2}$. The result is

(33) $$\begin{array}{cc} d\Delta s_{\mathrm{tot}} & =-d\ln p_{1}(x_{1},t)-\frac{1}{D_{11}}\frac{dD_{11}}{dx_{1}}dx_{1}\\ \; & -D_{11}\frac{d}{dx_{1}}\left(\frac{1}{D_{11}}\frac{dD_{11}}{dx_{1}}\right)dt\\ \; & =-d\ln p_{1}(x_{1},t)+2(1+x_{1}^{2})dt+4x_{1}dW. \end{array}$$

However, we could just as well decide to compute the stochastic entropy production by regarding $x_{2}$ as the dynamical variable and $x_{1}$ as the spectator and write

(34) $$\begin{array}{cc} & d\Delta s_{\mathrm{tot}}=-d\ln p_{2}(x_{2},t)+\frac{1}{D_{22}}A_{2}dx_{2}-\frac{1}{D_{22}}\frac{dD_{22}}{dx_{2}}dx_{2}\\ \; & +\left[D_{22}\frac{d}{dx_{2}}\left(\frac{1}{D_{22}}A_{2}\right)-D_{22}\frac{d}{dx_{2}}\left(\frac{1}{D_{22}}\frac{dD_{22}}{dx_{2}}\right)\right]dt, \end{array}$$

with $p_{2}(x_{2},t)=\int p(x_{1},x_{2},t)dx_{1}$, $A_{2}=-\frac{1}{2}x_{2}$ and $D_{22}=\frac{1}{2}x_{1}^{2}x_{2}^{2}$. Since $D_{22}$ depends on the (currently chosen) spectator variable $x_{1}$, we have to employ the derivative

(35) $$\frac{dD_{22}}{dx_{2}}=\frac{\partial D_{22}}{\partial x_{2}}+R\frac{\partial D_{22}}{\partial x_{1}},$$

and identify the coefficient *R* using the (single) null eigenvector of D, which may be shown to be given by ${\left(x_{1}/x_{2}^{2},(1-x_{1}^{2})/x_{2}^{3}\right)}^{T}$.

**Figure 2:**
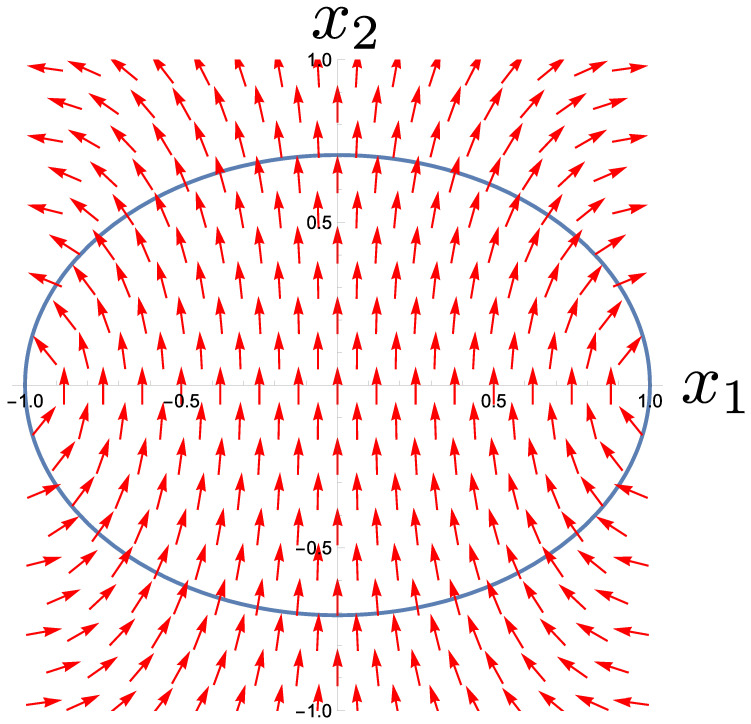
The system discussed in Section 4 performs diffusion that is restricted to an elliptical contour shown in blue, with fixed point attractors at $x_{1}=\pm 1$ and $x_{2}=0$. The directions of the null eigenvectors of the diffusion matrix are shown at points in the space to demonstrate that they lie locally normal to the contour.

In this example, the dynamics preserve the value of the function $f(x_{1},x_{2})=\left(x_{1}^{2}+Kx_{2}^{2}-1\right)/(2x_{2}^{3})=0$ with arbitrary constant *K* (i.e., the motion is confined to an elliptical contour centered on the origin). The phase space for the restricted diffusion is illustrated in Figure 2 for K=2, together with a set of local null eigenvectors to show that they lie perpendicular to the contours. The stochastic dynamics take the system towards fixed point attractors at $x_{1}=\pm 1$ and $x_{2}=0$.

We can use Equation (14) in the form $\alpha_{1}dx_{1}+\alpha_{2}dx_{2}=0$, where $\alpha_{i}$ is the *i*th component of the null eigenvector. We then obtain

(36) $$x_{1}x_{2}dx_{1}=-\left(1-x_{1}^{2}\right)dx_{2},$$

such that $R=dx_{1}/dx_{2}=-(1-x_{1}^{2})/(x_{1}x_{2})$ and

(37) $$\frac{dD_{22}}{dx_{2}}=x_{1}^{2}x_{2}-\frac{\left(1-x_{1}^{2}\right)}{x_{1}x_{2}}x_{1}x_{2}^{2}=2x_{1}^{2}x_{2}-x_{2}.$$

The computation of $\Delta s_{\mathrm{tot}}$ using Equations (34) and (35) can then proceed, yielding

(38) $$\begin{array}{cc} d\Delta s_{\mathrm{tot}}= & -d\ln p_{2}(x_{2},t)+\frac{1}{2x_{1}^{2}}\left(1+x_{1}^{2}+4x_{1}^{4}\right)dt\\ \; & -\frac{1}{x_{1}}\left(1-4x_{1}^{2}\right)dW. \end{array}$$

The two SDEs, (33) and (38), describing the evolution of the total stochastic entropy production look different but will yield the same statistics. This can be demonstrated by writing $p_{1}(x_{1},t)dx_{1}=p_{2}(x_{2},t)dx_{2}$; hence,

(39) $$-d\ln p_{1}(x_{1},t)=-d\ln p_{2}(x_{2},t)+d\ln\left|R\right|,$$

and then we use the rules of stochastic calculus to show that $d\ln|R|=\frac{1}{2}\left(x_{1}^{-2}-3\right)dt-x_{1}^{-1}dW$.

The point we are making is that, in cases of restricted diffusive evolution, with more coordinates than independent noises, we can divide the variables arbitrarily into dynamical and spectator sets and use the former to calculate the stochastic entropy production. The implication is that some choices regarding the division might be more convenient than others. In the case just considered, it is more sensible to regard $x_{1}$ as the dynamical variable and $x_{2}$ as the spectator in order to avoid mathematical difficulties such as the divergence in the right-hand side of Equation (38) at $x_{1}=0$ or equivalently at $x_{2}=\pm \sqrt{K}$. Furthermore, the Fokker–Planck equation for the PDF $p_{1}(x_{1},t)$ is easy to construct,

(40) $$\frac{\partial p_{1}}{\partial t}=\frac{1}{2}\frac{\partial^{2}(1-x_{1}^{2})p_{1}}{\partial x_{1}^{2}},$$

while that for $p_{2}(x_{2},t)$ is much more difficult to analyze.

Having said this, it would not be sensible to choose as dynamical variables a set that evolves deterministically, without noise. The reduced diffusion matrix for such a set would vanish, and the calculation of the stochastic entropy production would not then be possible.

## 5. An Open Three-Level Quantum System with Restricted Diffusion

### 5.1. SDEs and Selection of Spectator Variables

We have developed the present framework for computing stochastic entropy production because there are physical systems of interest where some of the stochastically evolving variables are spectators. In this section, we consider the dynamics of an open quantum system characterized by the stochastic evolution of its reduced density matrix ρ, which is the appropriate representation of the state [10,11,12,13]. The stochasticity of the evolution can be interpreted as a consequence of the coupling to the environment. The stochastic entropy production associated with such evolution of the reduced density matrix has recently received attention [6,7,14,15]. It provides a measure of the irreversibility of open quantum system evolution and also characterizes the evolving uncertainty of the quantum state of a system and its environment under incompletely specified dynamics.

**Figure 3:**
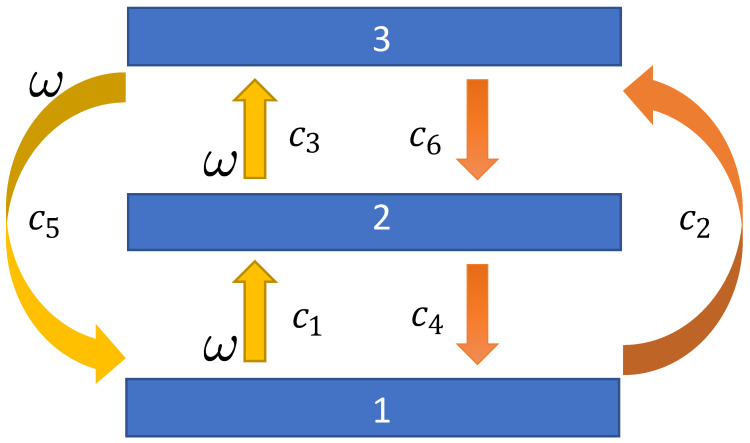
A sketch of the dynamics of an open three-level quantum system with system–environment coupling characterized by the set of six raising ($c_{1–3}$) and lowering ($c_{4–6}$) operators. By reducing the probabilities of the transitions shown in yellow, through choosing a value of the weighting factor ω<1 in the Lindblad operators $c_{1,3,5}$ in Equation (42), a probability current passing through states 1→3→2→1 and characterized by positive mean stochastic entropy production can be generated. For ω=1, the system would adopt an equilibrium state with zero current and zero stochastic entropy production. The adjustment of $c_{1,3,5}$ by way of the weighting factor ω is indicated in the sketch.

In Appendix B, it is shown how a Markovian stochastic Lindblad equation for the evolution of the reduced density matrix of an open system can be derived, starting from the Lindblad operators that specify the dynamical effects of the environment on the system. Using this formalism, we consider a three-level bosonic system with environmental coupling characterized by the three raising ($c_{1-3}$) and three lowering ($c_{4-6}$) Lindblad operators given by

(41) $$\begin{array}{cc} & c_{1}=\omega \left(\begin{array}{ccc} 0 & 0 & 0\\ 1 & 0 & 0\\ 0 & 0 & 0 \end{array}\right)\;\;c_{2}=\left(\begin{array}{ccc} 0 & 0 & 0\\ 0 & 0 & 0\\ 1 & 0 & 0 \end{array}\right)\;\;c_{3}=\omega \left(\begin{array}{ccc} 0 & 0 & 0\\ 0 & 0 & 0\\ 0 & 1 & 0 \end{array}\right)\\ \; & c_{4}=\left(\begin{array}{ccc} 0 & 1 & 0\\ 0 & 0 & 0\\ 0 & 0 & 0 \end{array}\right)\;\;c_{5}=\omega \left(\begin{array}{ccc} 0 & 0 & 1\\ 0 & 0 & 0\\ 0 & 0 & 0 \end{array}\right)\;\;c_{6}=\left(\begin{array}{ccc} 0 & 0 & 0\\ 0 & 0 & 1\\ 0 & 0 & 0 \end{array}\right) \end{array}$$

in a basis of kets |1〉,|2〉, and |3〉 corresponding to the three levels. The role of the constant ω is to induce and control a nonequilibrium stationary state in the system by the breakage of detailed balance, as will be described in Section 5.2. The SDE describing the dynamics of the system is given by the stochastic Lindblad equation

(42) $$\begin{array}{c} d\rho =\sum_{i=1}^{6}\left[\left(c_{i}\rho c_{i}^{†}-\frac{1}{2}\rho c_{i}^{†}c_{i}-\frac{1}{2}c_{i}^{†}c_{i}\rho \right)dt\;\;\;\;\;\;\;\;+(\rho c_{i}^{†}+c_{i}\rho -\mathrm{Tr}[\rho (c_{i}+c_{i}^{†})]\rho )dW_{i}\right], \end{array}$$

and a sketch of the inter-level transitions brought about by the $c_{i}$ is given in Figure 3.

The reduced density matrix ρ for this system is a complex 3×3 Hermitian matrix with a unit trace, representing eight degrees of freedom. We therefore parameterize ρ in terms of an eight-dimensional vector x with components $x_{i}$, evolving as

(43) $$dx_{i}=A_{i}dt+\sum_{j}B_{ij}dW_{j},$$

in terms of an eight-dimensional vector A, an 8×6 matrix B, and a six-dimensional vector dW of independent Wiener increments. Since there are fewer noise terms than SDEs, the diffusive motion will be restricted in some way.

In order to proceed, we represent the density matrix using the eight Gell–Mann matrices given by

(44) $$\begin{array}{cc} & \lambda_{1}=\left(\begin{array}{ccc} 0 & 1 & 0\\ 1 & 0 & 0\\ 0 & 0 & 0 \end{array}\right)\;\lambda_{2}=\left(\begin{array}{ccc} 0 & -i & 0\\ i & 0 & 0\\ 0 & 0 & 0 \end{array}\right)\;\lambda_{3}=\left(\begin{array}{ccc} 1 & 0 & 0\\ 0 & -1 & 0\\ 0 & 0 & 0 \end{array}\right)\\ \; & \lambda_{4}=\left(\begin{array}{ccc} 0 & 0 & 1\\ 0 & 0 & 0\\ 1 & 0 & 0 \end{array}\right)\;\lambda_{5}=\left(\begin{array}{ccc} 0 & 0 & -i\\ 0 & 0 & 0\\ i & 0 & 0 \end{array}\right)\;\lambda_{6}=\left(\begin{array}{ccc} 0 & 0 & 0\\ 0 & 0 & 1\\ 0 & 1 & 0 \end{array}\right)\\ \; & \lambda_{7}=\left(\begin{array}{ccc} 0 & 0 & 0\\ 0 & 0 & -i\\ 0 & i & 0 \end{array}\right)\;\lambda_{8}=\frac{1}{\sqrt{3}}\left(\begin{array}{ccc} 1 & 0 & 0\\ 0 & 1 & 0\\ 0 & 0 & -2 \end{array}\right). \end{array}$$

It is convenient to work with eight real variables s,r,u,v,w,x,y, and *z*, defined by $\mathrm{Tr}(\lambda_{1}\rho )=s$, $\mathrm{Tr}(\lambda_{2}\rho )=r$, $\mathrm{Tr}(\lambda_{3}\rho )=u$, etc. We can then write ρ as

(45) $$\rho =\left(\begin{array}{ccc} \frac{u}{2}+\frac{\sqrt{3}z}{6}+\frac{1}{3} & \frac{s}{2}-\frac{ir}{2} & \frac{v}{2}-\frac{iw}{2}\\ \frac{s}{2}+\frac{ir}{2} & -\frac{u}{2}+\frac{\sqrt{3}z}{6}+\frac{1}{3} & \frac{x}{2}-\frac{iy}{2}\\ \frac{v}{2}+\frac{iw}{2} & \frac{x}{2}+\frac{iy}{2} & -\frac{\sqrt{3}z}{3}+\frac{1}{3} \end{array}\right).$$

The SDEs describing the evolution of the eight stochastic variables with, for simplicity, weighting factor ω=1, are given in Appendix C.

We choose *y* and *z* as spectator variables, allowing us to focus instead on the six SDEs given by

(46) $$\left(\begin{array}{c} ds\\ dr\\ du\\ dv\\ dw\\ dx \end{array}\right)=\left(\begin{array}{c} -2s\\ -2r\\ -3u\\ -2v\\ -2w\\ -2x \end{array}\right)dt+\left(\begin{array}{cccccc} -sv+x & -s^{2}-u+\frac{\sqrt{3}z}{3}+\frac{2}{3} & -sx+v & -sv & -s^{2}+u+\frac{\sqrt{3}z}{3}+\frac{2}{3} & -sx\\ -rv-y & -sr & -rx+w & -rv & -sr & -rx\\ v(1-u) & s(1-u) & -x(u+1) & -uv & -s(u+1) & -ux\\ -v^{2}-\frac{2\sqrt{3}z}{3}+\frac{2}{3} & -sv+x & -vx & u-v^{2}+\frac{\sqrt{3}z}{3}+\frac{2}{3} & -sv & s-vx\\ -vw & -sw+y & -wx & -vw & -sw & r-wx\\ -vx & -sx & -x^{2}-\frac{2\sqrt{3}z}{3}+\frac{2}{3} & s-vx & -sx+v & -u-x^{2}+\frac{\sqrt{3}z}{3}+\frac{2}{3} \end{array}\right)\left(\begin{array}{c} dW_{1}\\ dW_{2}\\ dW_{3}\\ dW_{4}\\ dW_{5}\\ dW_{6} \end{array}\right).$$

The reduced diffusion matrix that arises from Equation (46) is too elaborate to obtain an analytical expression for its inverse. It is possible, however, to compute an inverse numerically and then to append terms to any derivatives of the matrix elements according to the procedure described in Section 3. In actual fact, with this choice in spectator variables, *no* such additional terms are required, making this route very convenient.

### 5.2. Equilibrium and Nonequilibrium Stationary States

We have solved the SDEs for the dynamics of the reduced density matrix numerically (using a simple Euler–Maruyama scheme) and computed the evolution of the environmental component $\Delta s_{\mathrm{env}}$ of the stochastic entropy production, introduced in Equation (5). Calculating the system component $\Delta s_{\mathrm{sys}}$ requires a solution of a Fokker–Planck equation, which is computationally demanding, and doing so does not add to the understanding we aim to develop regarding the *stationary* states of the system, so we concentrate our attention instead on the environmental component. We comment further on this point later.

The computational demands of solving Equation (6) are considerable for the system under investigation, so only limited ensembles of trajectories were generated. Runs for environmental stochastic entropy production were typically executed with a timestep of $dt=10^{-6}$, which proved to be small enough not to have a noticeable effect on the statistics of the resulting ensemble of trajectories. The reduced density matrix was initiated in the condition where all variables *s*, etc., are set to 0.1.

**Figure 4:**
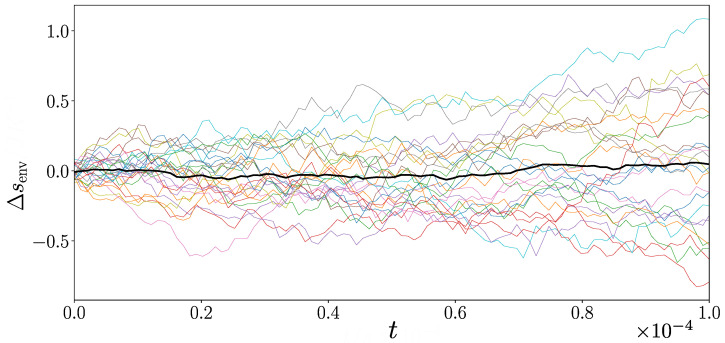
Environmental component of stochastic entropy production $\Delta s_{\mathrm{env}}$ as a function of time for the three-level quantum system, computed from 25 trajectories, each in a different colour, with equally weighted Lindblad operators (i.e., ω=1). Both $\Delta s_{\mathrm{env}}$ and time *t* are in dimensionless units. The black line represents the ensemble mean and is consistent with zero, as is to be expected in an equilibrium state.

Figure 4 illustrates the range of environmental stochastic entropy production for 25 runs with ω=1. The average environmental stochastic entropy production is nearly always within one standard deviation of zero, which, given the limited statistics, provides a strong indication that a zero mean rate of environmental stochastic entropy production has been established in the stationary state. This is precisely what is to be expected of an equilibrium state. The mean rate of system stochastic entropy production is expected to be zero as well since it corresponds in typical situations to the rate of change of the Gibbs entropy of the system [7], which characterizes the uncertainty in its adopted state, and which does not change in a stationary state.

It is worth mentioning here that we take the view that the system (either on its own or in association with the environment) does indeed adopt a particular quantum state at all times. We further regard the measurement of a system observable as a dynamical process whereby the state is driven towards specific stationary outcomes, the eigenstates of the observable [6,7,10,14,15,16]. The uncertainty in the outcome under ‘projective’ measurement for a given density matrix is the von Neumann entropy, which we discuss in Section 5.3. In contrast, the Gibbs entropy arising from our application of stochastic thermodynamics to the quantum system characterizes instead the uncertainty in the pre-measurement state. It therefore has quite a different scale since the range of possibilities is greater.

It is also worth mentioning that the treatment of stochastic entropy production in open quantum system dynamics described here differs from other approaches where instantaneous projective measurement is explicitly included in the dynamical scheme and where initial and final states for trajectories are restricted to measured eigenstates [17,18,19,20,21]. Our approach considers more general evolutions and a more general entropy, as already mentioned.

We now investigate a nonequilibrium stationary state with a probability current passing through the system phase space. Our interest in the three-level system arises precisely because it is the simplest quantum system in which such a situation might be possible. We create a nonequilibrium stationary state by breaking detailed balance and favoring a pattern of transitions |3〉→|2〉→|1〉→|3〉 around the system. We achieve this by reducing the coupling strength associated with the Lindblad operators linked to the opposite pattern. Specifically, we set the weighting factor ω<1 in Lindblad operators $c_{1}$, $c_{2}$, and $c_{5}$ in (41) and derive a modified set of dynamical equations. Such a nonequilibrium stationary state is expected to be associated with a positive mean rate of stochastic entropy production.

**Figure 5:**
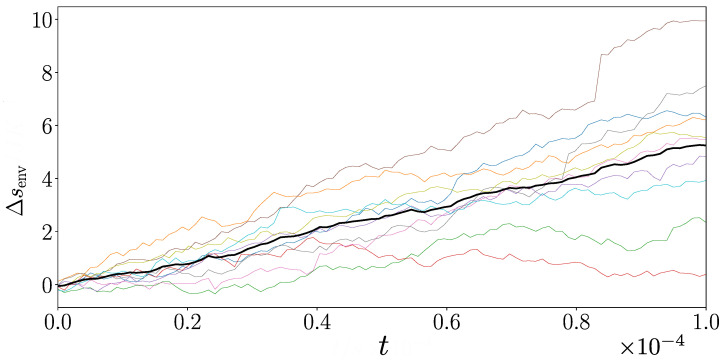
Environmental stochastic entropy production $\Delta s_{\mathrm{env}}$ in the three-level system computed for 10 trajectories, each in a different colour, with ω=0.1 and $dt=10^{-6}$. Both $\Delta s_{\mathrm{env}}$ and time *t* are in dimensionless units. The black line represents the ensemble mean.

Figure 5 illustrates the environmental stochastic entropy production for an ensemble of 10 runs with ω=0.1. In line with expectations, we see a constant mean rate of environmental stochastic entropy production associated with the system being in a nonequilibrium stationary state. The mean *system* component of stochastic entropy production is not expected to make a contribution in a stationary state since the Gibbs entropy of the system is constant in time for such a situation. There is a clear relationship between the breakage of detailed balance and the mean rate of environmental stochastic entropy production. Figure 5 provides strong support that our approach can quantify the irreversibility of open quantum systems with spectator variables.

### 5.3. Stochastic Entropy Production in Quantum Mechanics

The mean stochastic entropy production in the example just considered is the change in subjective uncertainty with regard to the quantum state adopted by the system and its environment (the world). We are unable to make exact predictions when the dynamics are not specified in detail: under an evolution such as Equation (42), our knowledge of the adopted state is therefore reduced with time. We should contrast this with the von Neumann entropy $S_{\mathrm{vN}}=-\mathrm{Tr}\rho \ln\rho$, a commonly employed expression for entropy in quantum mechanics.

Von Neumann entropy is the uncertainty with regard to the outcomes of projective measurement of a system. It is a Shannon entropy $-\sum_{i}P_{i}\ln P_{i}$, where $P_{i}$ is the probability of projection into eigenstate *i* of the observable. For an *N*-level system, the number of such outcomes is *N*, so the von Neumann entropy has an upper limit of lnN.

In contrast, there is no upper limit of the mean stochastic entropy production under general dynamics since there is a continuum of potentially adoptable quantum states of the world. The nonzero mean rate of production of stochastic entropy associated with the nonequilibrium stationary state considered in the last section represents this progressively greater uncertainty.

It is worth drawing this distinction since different probability density functions for the reduced density matrix of a system could have the same probabilities of projective measurement and hence the same von Neumann entropy and yet would express different uncertainties with regard to the adopted state prior to measurement, and hence different Gibbs entropies over the phase space of reduced density matrix elements.

Note also that the stochastic entropy production we have been considering has no connection with heat transfer or work. The three-level system under consideration does not possess an internal Hamiltonian *H*, and there is no change in system internal energy. Stochastic entropy production is not necessarily associated with the dissipation of potential energy into heat. In both classical and quantum settings, the purpose of entropy is to specify the degree of configurational uncertainty of a system. In classical mechanics, the configurations are described by sets of classical coordinates. In quantum mechanics, they are specified by collections of (reduced) density matrix elements.

## 6. Conclusions

We have employed Itô processes to model the dynamics and thermodynamics of a system interacting with an environment when detailed information about the exact configuration of either is lacking. Such an approach has frequently been used in situations described by classical dynamics [3], and also for evolving quantum systems [6,7,17,19]. In both cases, difficulties can arise when there are fewer independent sources of noise than dimensions of the system phase space. Diffusion is restricted and the diffusion matrix becomes singular, which complicates the calculation of stochastic entropy production.

The solution to the problem is simply to eliminate degrees of freedom from the entropy calculation to account for the existence of functions of the coordinates that evolve without noise. We have described a general method for doing so and illustrated it for a simple system of particle diffusion on an ellipse with two fixed points. We divide the stochastic variables into dynamical and spectator sets and construct a reduced nonsingular diffusion matrix and the associated dynamics of stochastic entropy production.

We have gone on to consider a three-level quantum system, thermalized by an environment that brings about transitions between the levels. The dynamics take place in an eight-dimensional phase space with only six noise terms, and the 8×8 diffusion matrix is singular.

We have shown that an equilibrium state of the system may be established, characterized by a zero mean rate of environmental stochastic entropy production (and implicitly by a zero mean rate of system stochastic entropy production as well). More interestingly, we have adjusted the probabilities of the transitions induced by the environment to create a nonequilibrium stationary state, where the particle is made to cycle, on average, through the levels in a particular order. This state is characterized by a nonzero rate of mean environmental stochastic entropy production.

Stochastic entropy production realizes Boltzmann’s aim of linking thermodynamics, and specifically entropy production, to the dynamical evolution of system coordinates [22]. Entropy production quantifies the irreversibility of open system behavior, assessing the likelihood that reversals of sequences of events might be observed. Refining this framework to accommodate special cases such as restricted diffusion adds further scope for quantifying irreversibility in open classical and quantum systems.

## Data Availability

The original contributions presented in this study are included in the article. Further inquiries can be directed to the corresponding author.

## Appendix A. General Expression for Environmental Stochastic Entropy Production

An elaborate framework is needed if some of the dynamical variables are odd under time reversal symmetry. The deterministic terms in the SDEs need to be separated into reversible and irreversible components [5]:

(A1) $$\begin{array}{ccc} A_{i}^{\mathrm{irr}}(x) & = & \frac{1}{2}\left[A_{i}(x)+\varepsilon_{i}A_{i}(\varepsilon x)\right]=\varepsilon_{i}A_{i}^{\mathrm{irr}}(\varepsilon x),\; \end{array}$$

and

(A2) $$A_{i}^{\mathrm{rev}}(x)=\frac{1}{2}\left[A_{i}(x)-\varepsilon_{i}A_{i}(\varepsilon x)\right]=-\varepsilon_{i}A_{i}^{\mathrm{rev}}(\varepsilon x),$$

where $\varepsilon_{i}=1$ for variables $x_{i}$ with even parity under time reversal symmetry (for example, position) and $\varepsilon_{i}=-1$ for variables with odd parity (for example, velocity). The form εx represents $\left(\varepsilon_{1}x_{1},\varepsilon_{2}x_{2},\cdots \right)$. As the notation suggests, the $A_{i}^{\mathrm{irr}}$ terms are partially responsible for irreversible behavior, while the $A_{i}^{\mathrm{rev}}$ terms are time reversal invariant and are not responsible. The evolution of the environmental stochastic entropy production is then governed by [5]

(A3) $$\begin{array}{cc} & d\Delta s_{\mathrm{env}}=-\sum_{i}\frac{\partial A_{i}^{\mathrm{rev}}}{\partial x_{i}}dt+\sum_{i,j}\{D_{ij}^{-1}A_{i}^{\mathrm{irr}}dx_{j}\\ \; & -D_{ij}^{-1}\sum_{m}\frac{\partial D_{im}}{\partial x_{m}}dx_{j}-D_{ij}^{-1}A_{i}^{\mathrm{rev}}A_{j}^{\mathrm{irr}}dt\\ \; & +D_{ij}^{-1}A_{i}^{\mathrm{rev}}\sum_{n}\frac{\partial D_{jn}}{\partial x_{n}}dt+\sum_{k}[D_{ik}\frac{\partial }{\partial x_{k}}\left(D_{ij}^{-1}A_{j}^{\mathrm{irr}}\right)\\ \; & -D_{ik}\frac{\partial }{\partial x_{k}}\left(D_{ij}^{-1}\sum_{n}\frac{\partial D_{jn}}{\partial x_{n}}\right)]dt\}. \end{array}$$

For a system described by even parity variables with $\varepsilon_{i}=1$, the reversible terms $A_{i}^{\mathrm{rev}}$ are all zero and $A_{i}^{\mathrm{irr}}=A_{i}$. Equation (A3) then simplifies to

(A4) $$\begin{array}{cc} & d\Delta s_{\mathrm{env}}=\sum_{i,j}\{D_{ij}^{-1}A_{i}dx_{j}-D_{ij}^{-1}\sum_{m}\frac{\partial D_{im}}{\partial x_{m}}dx_{j}+\\ \; & \sum_{k}\left[D_{ik}\frac{\partial }{\partial x_{k}}\left(D_{ij}^{-1}A_{j}\right)-D_{ik}\frac{\partial }{\partial x_{k}}\left(D_{ij}^{-1}\sum_{m}\frac{\partial D_{jm}}{\partial x_{m}}\right)\right]dt\}. \end{array}$$

which then takes the compact form of Equation (6).

## Appendix B. SDEs for Quantum State Diffusion

The reduced density matrix ρ describing an open quantum system may be considered to evolve stochastically in a timestep dt according to quantum maps specified by Kraus operators [13], the details of which are provided here.

We consider *i* pairs of Kraus operators:

(A5) $$M_{i\pm }=\sqrt{\frac{P_{i}}{2}}\left(I-\frac{1}{2P_{i}}c_{i}^{†}c_{i}dt\pm \frac{1}{\sqrt{P_{i}}}c_{i}\sqrt{dt}\right),$$

where $c_{i}$ represents the Lindblad operators that specify the mode of coupling between the system and environment. The meaning of the $P_{i}$ will become clear shortly. A similar scheme has been outlined elsewhere [6,7,14], in which $P_{i}$ variables do not appear.

The Kraus operators satisfy

(A6) $$\begin{array}{c} M_{i\pm }^{†}M_{i\pm }=\frac{P_{i}}{2}\left(I\pm \frac{1}{\sqrt{P_{i}}}\left(c_{i}+c_{i}^{†}\right)\sqrt{dt}\right), \end{array}$$

so that $M_{i+}^{†}M_{i+}+M_{i-}^{†}M_{i-}=P_{i}I$, and the completeness relation [11]

(A7) $$\sum_{i}\left(M_{i+}^{†}M_{i+}+M_{i-}^{†}M_{i-}\right)=I,$$

therefore holds if $\sum_{i}P_{i}=1$.

Starting from ρ, a target set of 2i reduced density matrices $\rho^{\prime i\pm }$, all of which are positive definite and of unit trace, are taken to be reachable after a timestep dt through a map associated with one of the Kraus operators:

(A8) $$\rho^{\prime i\pm }=\frac{M_{i\pm }\rho M_{i\pm }^{†}}{\mathrm{Tr}(M_{i\pm }\rho M_{i\pm }^{†})}.$$

The probabilities of making the transitions to these targets are given by

(A9) $$\begin{array}{cc} p_{i\pm } & =\mathrm{Tr}(M_{i\pm }\rho M_{i\pm }^{†})\\ \; & =\frac{P_{i}}{2}\left(1\pm \sqrt{\frac{dt}{P_{i}}}\mathrm{Tr}\left[(c_{i}+c_{i}^{†})\rho \right]\right), \end{array}$$

which satisfies $p_{i+}+p_{i-}=P_{i}$ and $p_{i+}-p_{i-}=P_{i}\sqrt{\frac{dt}{P_{i}}}\mathrm{Tr}\left[(c_{i}+c_{i}^{†})\rho \right]$. $P_{i}$ is therefore taken to be the probability that one of the *i*th pairs of Kraus operators is selected for the map: it is the probability that the environment disturbs the system in such a way as to transform ρ into either $\rho^{\prime i+}$ or $\rho^{\prime i-}$. As a consequence of these dynamical rules, the reduced density matrix is driven along a Brownian trajectory under the influence of the environment. Since the Kraus operators reduce to a multiple of the identity as dt→0, the Brownian path is continuous. No finite quantum jumps are considered (and neither are they necessary [7]).

The statistically averaged form of the new reduced density matrix is

(A10) $$\begin{array}{cc} \langle \rho^{\prime }\rangle & =\sum_{i}\left(p_{i+}\frac{M_{i+}\rho M_{i+}^{†}}{\mathrm{Tr}(M_{i+}\rho M_{i+}^{†})}+p_{i-}\frac{M_{i-}\rho M_{i-}^{†}}{\mathrm{Tr}(M_{i-}\rho M_{i-}^{†})}\right)\\ \; & =\sum_{i}\left(M_{i+}\rho M_{i+}^{†}+M_{i-}\rho M_{i-}^{†}\right), \end{array}$$

which corresponds in notation with the standard Kraus map [13].

The average evolution having been established, we now derive an SDE that describes the *stochastic* evolution of the reduced density matrix. The possible increments after the timestep are $d\rho^{i\pm }=\rho^{\prime i\pm }-\rho$, where

(A11) $$\begin{array}{cc} d\rho^{i\pm } & =\frac{1}{P_{i}}\left(c_{i}\rho c_{i}^{†}-\frac{1}{2}\rho c_{i}^{†}c_{i}-\frac{1}{2}c_{i}^{†}c_{i}\rho \right)dt\\ - & \frac{1}{P_{i}}\left(\rho c_{i}^{†}+c_{i}\rho -\mathrm{Tr}\left[\rho (c_{i}+c_{i}^{†})\right]\rho \right)\mathrm{Tr}\left[\rho (c_{i}+c_{i}^{†})\right]dt\\ \pm & \frac{1}{\sqrt{P_{i}}}\left(\rho c_{i}^{†}+c_{i}\rho -\mathrm{Tr}\left[\rho (c_{i}+c_{i}^{†})\right]\rho \right)\sqrt{dt}. \end{array}$$

The average increment in the reduced density matrix is then

(A12) $$\langle d\rho \rangle =\sum_{i}\left(p_{i+}d\rho^{i+}+p_{i-}d\rho^{i-}\right),$$

which leads after some manipulation to

(A13) $$\langle d\rho \rangle =\sum_{i}\left(c_{i}\rho c_{i}^{†}-\frac{1}{2}\rho c_{i}^{†}c_{i}-\frac{1}{2}c_{i}^{†}c_{i}\rho \right)dt.$$

The terms in the sum are the dissipators associated with each Lindblad operator, in the form in which they appear in the standard Lindblad equation [13].

Next, we compute the variance of dρ. For clarity, we write

(A14) $$d\rho^{i\pm }=\frac{1}{P_{i}}A_{i}dt\pm \frac{1}{\sqrt{P_{i}}}B_{i}\sqrt{dt},$$

where

(A15) $$\begin{array}{cc} A_{i} & =c_{i}\rho c_{i}^{†}-\frac{1}{2}\rho c_{i}^{†}c_{i}-\frac{1}{2}c_{i}^{†}c_{i}\rho \\ \; & -\left(\rho c_{i}^{†}+c_{i}\rho -\mathrm{Tr}\left[\rho (c_{i}+c_{i}^{†})\right]\rho \right)\mathrm{Tr}\left[\rho (c_{i}+c_{i}^{†})\right], \end{array}$$

and $B_{i}=\rho c_{i}^{†}+c_{i}\rho -\mathrm{Tr}\left[\rho (c_{i}+c_{i}^{†})\right]\rho$. We then construct the average of ${\left(d\rho^{i\pm }-\langle d\rho \rangle \right)}^{2}$ to lowest order in dt. The $A_{i}$ and 〈dρ〉 terms do not contribute because they are already of order dt, and we obtain

(A16) $$\begin{array}{c} \sum_{i}\left(p_{i+}{\left(d\rho^{i+}-\langle d\rho \rangle \right)}^{2}+p_{i-}{\left(d\rho^{i-}-\langle d\rho \rangle \right)}^{2}\right)\\ =\sum_{i}\left(p_{i+}\frac{1}{P_{i}}B_{i}^{2}dt+p_{i-}\frac{1}{P_{i}}B_{i}^{2}dt\right)=\sum_{i}B_{i}^{2}dt, \end{array}$$

suggesting that the Itô process governing the evolution of ρ is

(A17) $$\begin{array}{cc} d\rho & =\langle d\rho \rangle +\sum_{i}B_{i}dW_{i}\\ \; & =\sum_{i}\left[\left(c_{i}\rho c_{i}^{†}-\frac{1}{2}\rho c_{i}^{†}c_{i}-\frac{1}{2}c_{i}^{†}c_{i}\rho \right)dt\right.\\ \; & +\left(\rho c_{i}^{†}+c_{i}\rho -\mathrm{Tr}\left[\rho (c_{i}+c_{i}^{†})\right]\rho \right)dW_{i}], \end{array}$$

and this is the form employed in Equation (42).

The Kraus operators in Equation (A5) that underpin our framework are specified in terms of $P_{i}$ parameters that are to be interpreted as the probabilities of selection of one of the *i*th pairs of Kraus operators for the transformation of the reduced density matrix. By design, these parameters do not appear in the SDE, but it is worth discussing how they might be chosen for use in a numerical simulation, for example. If there are *N* Lindblad operators, a possible choice could be $P_{i}=1/N$. This is not entirely satisfactory, however, since it would assign equal selection weight to the Kraus operators irrespective of the degree of coupling between the system and its environment for each Lindblad operator. It would be better if we could ascribe a zero probability of selection to a Lindblad with zero coupling strength.

A solution would be to make $P_{i}$ dependent on the norm of the matrix representing the Lindblad operator. We could use the Frobenius norm ${∥c_{i}∥}_{F}=\sqrt{\mathrm{Tr}(c_{i}^{†}c_{i})}$, for example, and employ probabilities of selection

(A18) $$P_{i}=\frac{∥c_{i}{∥}_{F}}{\sum_{i}∥c_{i}{∥}_{F}}.$$

Adding a Lindblad $c_{i}=0$ to the set would not change the Kraus operators representing the other Lindblads, and, furthermore, such a Lindblad would have zero probability of being selected. The matter is somewhat academic since $P_{i}$ does not affect the derived form of the stochastic dynamics by design. It does, however, impact the elegance of the framework that emerges for modeling the diffusion of the quantum state.

## Appendix C. SDEs for the Three-Level System

The stochastic Lindblad equation Equation (42) with Lindblad operators (41) and density matrix parametrized in Equation (45) leads to the SDEs:

(A19) $$\left(\begin{array}{c} ds\\ dr\\ du\\ dv\\ dw\\ dx\\ dy\\ dz \end{array}\right)=\left(\begin{array}{c} -2s\\ -2r\\ -3u\\ -2v\\ -2w\\ -2x\\ -2y\\ -3z \end{array}\right)dt+\left(\begin{array}{cccccc} -sv+x & -s^{2}-u+\frac{\sqrt{3}z}{3}+\frac{2}{3} & -sx+v & -sv & -s^{2}+u+\frac{\sqrt{3}z}{3}+\frac{2}{3} & -sx\\ -rv-y & -sr & -rx+w & -rv & -sr & -rx\\ v(1-u) & s(1-u) & -x(u+1) & -uv & -s(u+1) & -ux\\ -v^{2}-\frac{2\sqrt{3}z}{3}+\frac{2}{3} & -sv+x & -vx & u-v^{2}+\frac{\sqrt{3}z}{3}+\frac{2}{3} & -sv & s-vx\\ -vw & -sw+y & -wx & -vw & -sw & r-wx\\ -vx & -sx & -x^{2}-\frac{2\sqrt{3}z}{3}+\frac{2}{3} & s-vx & -sx+v & -u-x^{2}+\frac{\sqrt{3}z}{3}+\frac{2}{3}\\ -vy & -sy & -xy & -r-vy & -sy+w & -xy\\ \frac{v(-3z+\sqrt{3})}{3} & \frac{s(-3z+\sqrt{3})}{3} & \frac{x(-3z+\sqrt{3})}{3} & -\frac{v(3z+2\sqrt{3})}{3} & \frac{s(-3z+\sqrt{3})}{3} & -\frac{x(3z+2\sqrt{3})}{3} \end{array}\right)\left(\begin{array}{c} dW_{1}\\ dW_{2}\\ dW_{3}\\ dW_{4}\\ dW_{5}\\ dW_{6} \end{array}\right).$$

The dynamics of ρ are therefore expressed in terms of N=8 Itô processes with M=6 noise terms. We expect the diffusion matrix $D=\frac{1}{2}BB^{T}$ to be singular (this can be checked using Mathematica) and for there to exist L=N−M=2 deterministically evolving functions of the stochastic variables s,⋯,z.
